# A Novel Nomogram Integrating Systemic Immune-Inflammation Index and Serum Prealbumin for Predicting Unplanned Readmission in Male Patients with Coexisting Lung Cancer and Chronic Obstructive Pulmonary Disease

**DOI:** 10.3390/cancers18050824

**Published:** 2026-03-04

**Authors:** Zhenjue Qian, Cuixia Niu, Jian Yang, Xingran Du, Yuting Wen, Li Wang, Hai Zhong, Xiuwei Zhang, Bing Wan, Zhangmin Ke

**Affiliations:** Department of Respiratory and Critical Care Medicine, Affiliated Jiangning Hospital of Nanjing Medicine University, Nanjing 211103, China

**Keywords:** lung cancer, COPD, readmission, nomogram

## Abstract

**Background:** Patients with coexisting lung cancer and COPD are highly susceptible to unplanned readmissions. This study aimed to develop and internally validate a robust predictive nomogram based on the “inflammation-nutrition-tumor” framework to quantify this risk. **Methods:** A retrospective cohort of 207 clinical episodes from male patients with lung cancer and COPD was analyzed. Participants were categorized into Planned Readmission (PR, *n* = 165) and Unplanned Readmission (UR, *n* = 42) groups. Independent risk factors were identified via univariate and multivariable analyses using Generalized Estimating Equations (GEE). A nomogram was subsequently constructed, and its performance was rigorously evaluated using the Area Under the Curve (AUC), calibration plots, and Decision Curve Analysis (DCA). **Results:** Multivariable GEE analysis demonstrated that the Systemic Immune-Inflammation Index (SII) was a highly significant independent risk factor (OR for a 500-unit increase = 1.490, 95% CI: 1.234–1.798, *p* < 0.001). Advanced cancer stage (III–IV) was also a significant predictor (OR = 3.590, 95% CI: 1.301–9.909, *p* = 0.014), while prealbumin (OR = 0.950, 95% CI: 0.896–1.007, *p* = 0.087) was identified as a key nutritional predictor. The integrated four-variable nomogram (age, cancer stage, SII, prealbumin) demonstrated good discriminative ability with an AUC of 0.809 (95% CI: 0.733–0.885). The calibration plot indicated excellent agreement, and DCA confirmed a substantial clinical net benefit. **Conclusions:** This SII-based nomogram provides a reliable and practical tool for individualized risk stratification, facilitating targeted clinical interventions to mitigate unplanned readmission rates in this vulnerable population.

## Simple Summary

Patients with coexisting lung cancer and chronic obstructive pulmonary disease (COPD) face a disproportionately high risk of unplanned readmission. This study developed and internally validated a user-friendly nomogram based on the “inflammation-nutrition-tumor” framework, using routinely available clinical parameters—specifically age, cancer stage, the Systemic Immune-Inflammation Index (SII), and serum prealbumin. SII is a composite marker derived from standard complete blood counts, reflecting systemic inflammation, while prealbumin serves as a sensitive indicator of nutritional status. The nomogram demonstrated good predictive accuracy (AUC = 0.809) and clinical utility in our male cohort, outperforming simpler models based on age and cancer stage alone. This tool may empower clinicians to identify high-risk patients and implement targeted, cost-effective interventions—such as nutritional optimization, intensified pulmonary rehabilitation, or closer post-discharge follow-up—with the goal of reducing avoidable readmissions and improving outcomes in this vulnerable population. External validation in diverse populations is planned before widespread clinical application.

## 1. Introduction

Lung cancer [1] and chronic obstructive pulmonary disease (COPD) [2,3] are the leading causes of respiratory-related mortality and disability worldwide. These two conditions are not only epidemiologically linked through shared risk factors such as tobacco smoking and environmental pollutants but are also pathophysiologically intertwined, forming a complex “lung cancer-COPD coexistence” [4]. Reports indicate that approximately 40–70% of lung cancer patients have comorbid COPD [5]. This coexistence creates a synergistic negative effect, which is associated with more severe clinical symptoms, limited treatment options, and significantly worsened prognosis compared to either disease alone. Notably, unplanned readmission has emerged as a critical negative indicator for healthcare quality and patient outcomes in this population [6,7,8].

Research suggests that nearly one-fifth of COPD patients are readmitted during the “vulnerable window” following discharge [9,10]. In patients where lung cancer and COPD coexist, this clinical management challenge is further amplified, as comorbid COPD is a significant risk factor for poor outcomes in lung cancer patients [11,12]. Frequent readmissions not only signal unsuccessful disease management but also impose heavy physical, psychological, and financial burdens on patients and their families while straining healthcare systems. Currently, clinical decisions regarding discharge readiness often rely on subjective experience or limited indicators, lacking quantified and personalized risk stratification tools for this specific comorbidity.

Traditionally, risk prediction has relied on comprehensive scoring systems such as the Charlson Comorbidity Index (CCI) or the Elixhauser Comorbidity Index [13,14,15]. However, these indices have notable limitations when applied to the unplanned readmission risk of lung cancer-COPD patients. The CCI, for instance, summarizes respiratory conditions as a binary “chronic pulmonary disease” category, failing to account for the severity of COPD (e.g., GOLD grades) or the dynamic exacerbation frequency [16,17]. Moreover, these static diagnostic counts cannot capture the acute pathophysiological changes occurring during the peri-discharge period.

Recent trends in precision medicine have shifted focus toward quantifiable biomarkers and pathophysiological indicators. Systemic inflammation and malnutrition are now recognized as pivotal factors influencing the prognosis of lung cancer patients [18,19]. Peripheral blood-derived inflammatory markers, such as the systemic immune-inflammation index (SII), offer a low-cost and highly accessible means of risk assessment [20]. Furthermore, integrating inflammatory markers with nutritional indicators like serum albumin and prealbumin has demonstrated superior prognostic value [21,22]. While nomograms have been utilized to visualize complex regression models for individualized risk assessment in various cancers [23,24,25,26,27,28,29], a specialized tool for predicting unplanned readmission in the lung cancer-COPD comorbid population remains scarce.

Therefore, the present study aimed to retrospectively evaluate the correlation between “inflammation-nutrition” axis indicators and unplanned readmission risk in patients with lung cancer and COPD. We sought to construct and internally validate a practical, visualized nomogram to facilitate identification and precise intervention for high-risk individuals, ultimately improving patient prognosis and optimizing healthcare resource allocation.

## 2. Materials and Methods

### 2.1. Study Design and Participant Selection

This retrospective study was conducted at Affiliated Jiangning Hospital of Nanjing Medical University. We identified patients diagnosed with both lung cancer and Chronic Obstructive Pulmonary Disease (COPD) between 1 January 2021 and 31 December 2025. Lung cancer was confirmed by pathological or cytological evidence, while COPD was diagnosed according to the Global Initiative for Chronic Obstructive Lung Disease (GOLD) criteria. The inclusion criteria were: (1) aged ≥ 18 years; (2) histologically confirmed lung cancer; (3) concomitant COPD. Exclusion criteria included: (1) major missing baseline clinical data; (2) presence of other active malignancies; (3) acute infections or systemic inflammatory diseases unrelated to lung cancer or COPD. A total of 207 (*n* = 207) qualified clinical episodes were finally recorded for analysis, with small amounts of missing prealbumin data handled through algorithmic imputation. Because this study investigates readmission, the first hospitalization for each patient was excluded from the analysis. Thus, all 207 episodes represent true readmission events. Notably, all patients identified during this period were male, thus the analysis was conducted on this male-specific cohort.

The study protocols adhered strictly to the ethical principles outlined in the Declaration of Helsinki and were approved by the Institutional Ethics Committee of Affiliated Jiangning Hospital of Nanjing Medicine University (Ethics Approval Number 2026-03-003-K01). Given the retrospective nature of the study, the requirement for informed consent was waived.

### 2.2. Data Acquisition and Variable Definitions

Comprehensive clinical data were systematically retrieved from the institutional electronic medical record (EMR) system. Demographic parameters included age and tobacco exposure history. Clinical and oncological variables encompassed cancer stage (classified according to the 8th edition of the American Joint Committee on Cancer TNM staging system), histological subtype, and COPD severity, graded per the GOLD criteria.

To ensure consistency and minimize the influence of acute perioperative inflammation or treatment-related fluctuations, all laboratory parameters were obtained from fasting venous blood samples collected during the initial clinical assessment within 24 h of admission, prior to any surgical intervention or systemic therapy. Systemic inflammatory biomarkers include absolute neutrophil count (ANC), absolute lymphocyte count (ALC), platelet count, and C-reactive protein (CRP). Nutritional and biochemical indices include serum albumin, serum prealbumin, and hemoglobin levels.

Specifically, the SII value was calculated using the standardized formula: $SII=(P_{0}\cdot N)/L$, where *P*_0_, *N*, and *L* represent platelet, ANC, and ALC (all expressed as 10^9^/L), respectively. Body Mass Index (BMI) was excluded from the final analysis due to a high proportion of inconsistent measurements and potential inter-observer variability during repeated admissions, which could compromise the stability and reliability of the predictive model. Prealbumin levels were missing in 9.7% (20/207) of cases. To assess the missing data pattern, we compared the clinical characteristics and primary outcomes between the missing group (*n* = 20) and the non-missing group (*n* = 187) for prealbumin. The results showed no significant difference in the primary outcome between the two groups (*p* = 0.973). Similarly, no significant differences were observed in cancer stage (*p* = 0.074), SII (*p* = 0.199), or serum albumin (*p* = 0.079). These findings suggest that the data are missing at random (MAR). Given the low proportion of missing data and its random nature, missing prealbumin values were handled using a median imputation algorithm. This sensitivity analysis confirmed that missing data were unlikely to introduce significant bias, as complete-case and imputed datasets yielded consistent results.

### 2.3. Definition of the Outcome

The primary outcome was unplanned readmission (UR), defined as any non-elective admission to our hospital within 90 days of discharge from the index hospitalization. We utilized an all-cause unplanned readmission definition to capture the overall clinical burden. A post hoc review indicated that the majority of these readmissions (approximately 90%) were directly attributable to acute exacerbations of COPD or complications from lung cancer (e.g., obstructive pneumonia, pleural effusion, or cancer-related pain). Conversely, planned readmissions (PR) were defined as scheduled, elective admissions for staged procedures or planned treatments and were excluded from the primary outcome analysis. Two clinicians independently reviewed the electronic health records to classify the nature of the readmissions, with a third senior oncologist acting as an adjudicator in cases of disagreement.

### 2.4. Statistical Analysis

Statistical processing was performed using R software version 4.5.2. Data normality was assessed for continuous variables using the Shapiro–Wilk test and visual inspection of Q-Q plots. Normally distributed continuous variables (e.g., hemoglobin, albumin) were reported as mean ± standard deviation (SD) and compared using Independent-samples *t*-test. Non-normally distributed data (e.g., SII, prealbumin) were expressed as medians and interquartile ranges Median (IQR) and analyzed using the Mann–Whitney *U* test. Categorical data were presented as frequencies (%) and analyzed using the $\chi^{2}$ test.

To identify robust predictors of readmission while accounting for the dependency among repeated observations from the same patient (207 episodes from 22 unique patients, with a median of 7 episodes per patient), univariate Generalized Estimating Equations (GEE) with a logit link function and an exchangeable working correlation structure were initially conducted. Variables exhibiting *p* < 0.10 were considered candidates for multivariable analysis. Adhering to the principle of parsimony and the clinical “inflammation-nutrition-tumor” hypothesis, a final multivariable GEE model comprising age, cancer stage, SII, and prealbumin was constructed. To evaluate the predictive value of the integrated biomarkers, a baseline model included only age and cancer stage was also built. A sensitivity analysis excluding the patient with the highest number of episodes (34 episodes) was also performed to assess the robustness of the findings.

A nomogram was subsequently developed based on the GEE model. Model performance was evaluated in terms of discrimination and calibration. Discriminative capacity was assessed using the Area Under the Receiver Operating Characteristic Curve (AUC). The difference in AUC between the proposed model and the baseline model was compared using the DeLong test. Model calibration was evaluated via calibration curves, where 207 episodes were divided into groups based on their predicted probabilities to compare the mean predicted probability with the actual observed readmission rate. Clinical utility was quantitatively assessed via Decision Curve Analysis (DCA). All statistical tests were two-sided, with *p* < 0.05 denoting statistical significance.

## 3. Results

### 3.1. Baseline Characteristics

A total of 207 clinical episodes with coexisting lung cancer and COPD were enrolled, comprising 165 (79.7%) in the PR group and 42 (20.3%) in the UR group. The number of episodes per patient ranged from 1 to 34, with a median of 7 (IQR: 3.25–14). The first hospitalization for each patient was excluded, consistent with this study’s focus on readmission. The comparative analysis of baseline clinical and laboratory parameters is detailed in Table 1. The median age of the entire cohort was 75 years [IQR: 69.0–78.0] in the PR group and 77 years [IQR: 71.0–78.3] in the UR group (*p* = 0.096).

Episodes in the UR group demonstrated a significantly heightened systemic inflammatory profile, characterized by markedly elevated SII levels [1474.5 (1110.1–1939.8) vs. 504.9 (344.9–712.5), *p* < 0.001] and CRP concentrations [19.7 (10.9–34.1) vs. 4.9 (2.1–10.4), *p* < 0.001]. Furthermore, nutritional depletion was more pronounced in the UR cohort, as evidenced by significantly lower serum albumin (34.3 ± 4.2 vs. 38.5 ± 3.9 g/L, *p* < 0.001) and prealbumin levels [159.5 (130.8–202.3) vs. 201.0 (161.0–246.0) mg/L, *p* = 0.002]. Oncologically, the UR group presented a higher prevalence of advanced-stage disease (Stage III–IV: 64.3% vs. 39.4%, *p* = 0.018) and increased COPD severity (GOLD III–IV: 35.7% vs. 20.0%, *p* = 0.031). Other parameters, including smoking history, histological subtype, and hemoglobin levels, showed no statistically significant disparities between the two groups (*p* > 0.05).

### 3.2. Predictor Selection and GEE Analysis Results

#### 3.2.1. Selection Strategy: Balancing Statistical Rigor and Clinical Utility

To develop a predictive model that is both statistically robust and clinically interpretable, we implemented a three-stage selection strategy focused on the “inflammation-nutrition” axis. First, all candidate covariates were initially evaluated using univariable GEE to identify potential predictors associated with the target outcome (*p* < 0.10), considering that a portion of patients experienced repeated readmissions. Second, for variables representing overlapping biological pathways, selection was guided by clinical sensitivity and physiological relevance. Specifically, between the inflammatory markers SII and CRP, SII was prioritized as it provides a more comprehensive reflection of the immune-inflammatory balance by integrating neutrophils, lymphocytes, and platelets. Similarly, prealbumin was favored over albumin due to its shorter half-life, making it a more sensitive indicator of acute nutritional fluctuations. Third, we compared the stability of models using different combinations of these indices. Variance Inflation Factors (VIF) were calculated to ensure the absence of significant multicollinearity among the final predictors.

#### 3.2.2. Results of Univariable and Multivariable GEE Analysis

Univariable GEE analysis identified several factors potentially associated with the outcome, including age (*p* = 0.055), cancer stage (*p* = 0.067), SII (*p* = 0.003), CRP (*p* < 0.001), prealbumin (*p* = 0.050), and albumin (*p* < 0.001). In the multivariable GEE analysis, when all six variables (age, cancer stage, SII, CRP, albumin, and prealbumin) were included, the *p*-values for SII and prealbumin were 0.384 and 0.369, respectively, with an AUC of 0.867. However, for the four variable model (age, cancer stage, SII, and prealbumin), the *p*-values for SII and prealbumin were <0.001 and 0.087, respectively. This sensitivity analysis confirms that CRP and albumin masked the independent contributions of SII and prealbumin, supporting our decision to retain the more clinically relevant markers in the final model. Consequently, the final model was constructed using age, cancer stage, SII, and prealbumin. The VIF values for age, cancer stage, SII, and prealbumin are 1.259, 1.112, 1.048, and 1.186, respectively, indicating no significant multicollinearity. The multivariable results demonstrated that higher age, advanced cancer stage, and elevated SII were independent risk factors, while higher prealbumin levels were associated with reduced risk. Detailed statistics are presented in Table 2. SII was treated as a continuous variable in increments of 500 units to facilitate a more clinically meaningful interpretation of the risk estimates. Consequently, the odds ratios (ORs) presented in Table 2 represent the change in the risk of UR for every 500-unit increase in the baseline SII level.

### 3.3. Construction and Performance Internal Validation of the Nomogram

**Figure 1:**
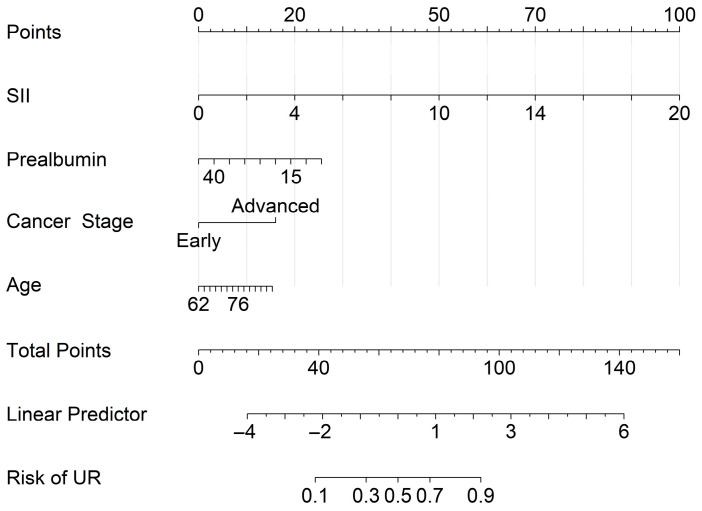
Nomogram for predicting the risk of unplanned readmission in male patients with coexisting lung cancer and COPD. To use the nomogram, locate the patient’s values for SII, prealbumin, cancer stage, and age on the corresponding axes. Note that the SII is plotted in increments of 500 units (10^9^/L) to provide a standardized contribution to the total score. Draw a vertical line upward to the “Points” axis to determine the points for each variable. Sum the points and locate the value on the “Total Points” axis. Draw a line downward to the “Risk of UR” axis to estimate the individualized risk.

Based on the independent predictors identified via multivariable GEE analysis, a visual predictive nomogram was developed (Figure 1). This tool translates the regression coefficients of age, cancer stage, SII, and prealbumin into a point-based scoring system. By summing the individual scores, clinicians can derive a personalized probability of unplanned readmission for each patient.

The model’s discriminative ability to distinguish between different outcomes was internally assessed using the AUC. In this cohort, the nomogram achieved an AUC of 0.809 (95% CI: 0.733–0.885), indicating strong discriminative performance. This was significantly higher than the baseline model (age and cancer stage only), which had an AUC of 0.677 (DeLong’s test, *p* = 0.0016). This sensitivity analysis demonstrates the incremental value of incorporating SII and prealbumin into the predictive model, confirming that the full nomogram provides superior risk stratification compared to basic clinical variables alone. Via the maximum Youden Index, the optimal cut-off value for the nomogram was determined as 0.210, yielding a sensitivity of 73.8% and a specificity of 79.3%. Another sensitivity analysis excluding the patient with the highest number of episodes (34 episodes) was conducted to assess the robustness of our findings. The results remained consistent: SII remained a strong independent predictor (OR = 1.548, 95% CI: 1.183–2.025, *p* = 0.001), and the model AUC improved slightly to 0.817 (95% CI: 0.737–0.896), confirming that our conclusions are not driven by a single outlier.

**Figure 2:**
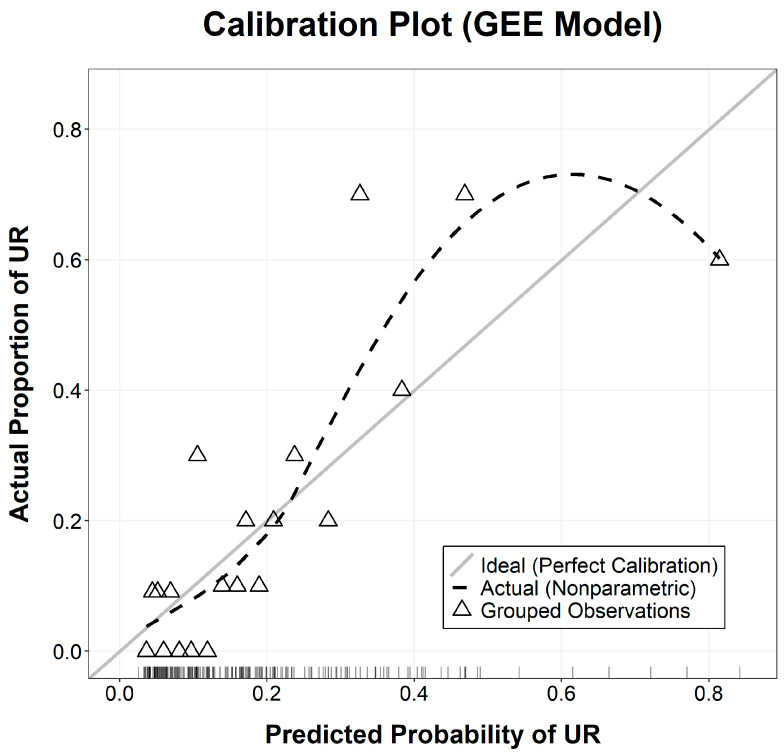
Calibration curve of the predictive nomogram. The X-axis represents the nomogram-predicted probability of unplanned readmission, and the Y-axis represents the actual observed probability. The Ideal line represents a perfect prediction where the predicted probability matches the actual outcome. The triangles represent the grouped observations (*n* = 20), where 207 episodes were divided into 20 nearly equal-sized groups based on predicted probabilities. The proximity of the triangles to the Ideal line indicates high calibration accuracy.

**Figure 3:**
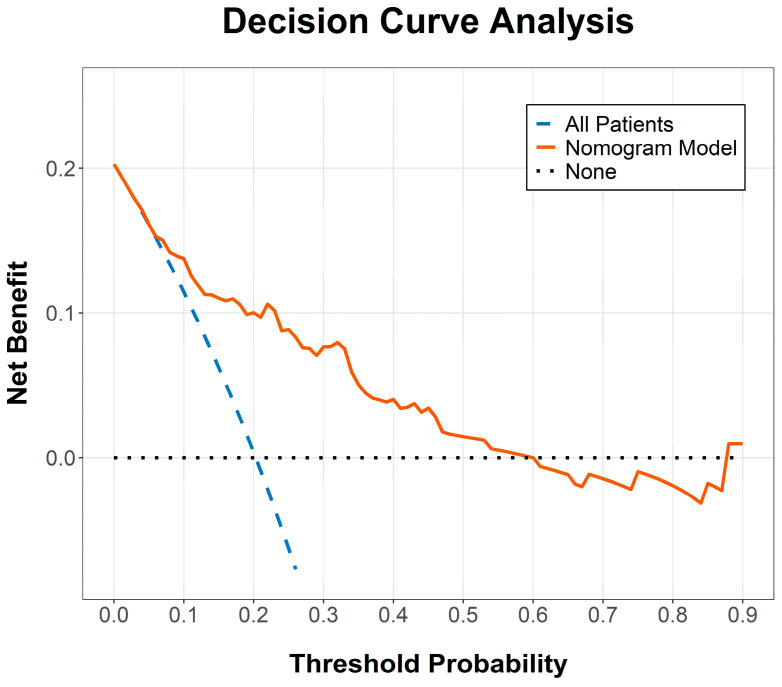
Decision curve analysis (DCA) for the integrated nomogram. The DCA evaluates the clinical net benefit of the predictive model. The Y-axis represents the net benefit, and the X-axis represents the threshold probability. The solid line represents the combined model (age + SII + prealbumin + cancer stage). The “All Patients” line assumes all patients will experience unplanned readmission, and the “None” line assumes no patients will. The gap between the red line and the two baseline lines indicates the clinical utility of the nomogram.

The calibration of the GEE-based model was evaluated using a calibration curve (Figure 2), where the 207 episodes were categorized into 20 nearly equal-sized groups based on their predicted probabilities. This approach ensured that each group contained approximately 10 to 11 cases. The curve demonstrated high agreement between the predicted probabilities and actual observed frequencies, with grouped data points closely aligning with the 45-degree Ideal line, indicating excellent calibration. Furthermore, DCA underscored the clinical utility of the model, demonstrating a superior net clinical benefit across a broad range of threshold probabilities (Figure 3). In this context, the threshold probability represents the level of risk at which a clinician would deem the benefits of an intervention (e.g., intensified monitoring) to outweigh the potential harms or costs. For instance, a threshold of 20% implies that the clinician considers the cost of a missed readmission to be four times greater than the cost of an unnecessary preventative intervention for a patient who would not have been readmitted. These findings confirm that the integrated nomogram is a reliable tool for identifying high-risk patients who may benefit from preemptive clinical management.

## 4. Discussion

### 4.1. The “Inflammation-Nutrition-Tumor” Axis in LC-COPD Coexistence

The current study successfully developed and internally validated a clinical nomogram aimed at predicting the risk of unplanned readmission in male patients with concurrent lung cancer and COPD. By integrating systemic inflammatory, nutritional, and oncological indices, we established a predictive tool with a good discriminative capacity (AUC = 0.809). Our findings underscore the pivotal role of the “inflammation-nutrition-tumor” axis in driving clinical instability in this specific patient population.

### 4.2. The Dominant Role of Systemic Inflammation (SII)

The most profound observation in our study was the strong predictive significance of SII. As a composite biomarker integrating neutrophil, lymphocyte, and platelet counts, SII provides a holistic perspective on the host’s immune-inflammatory equilibrium. In patients with lung cancer and COPD, elevated SII levels may reflect underlying chronic systemic inflammation—a common pathological substrate for both diseases. Neutrophils are known to play a role in tumor invasion and airway remodeling through the release of proteolytic enzymes, while lymphopenia has been associated with impaired anti-tumor immunity. Our multivariable analysis demonstrated that SII serves as a highly significant independent risk factor (*p* < 0.001), suggesting that intensified inflammatory monitoring could be instrumental in identifying patients prone to acute post-discharge deterioration.

### 4.3. Nutritional Vulnerability and the Significance of Prealbumin

Nutritional status, represented here by serum prealbumin, emerged as another critical component of our predictive framework. Although prealbumin did not meet the conventional threshold for statistical significance in the multivariable GEE model (*p* = 0.087), it was retained due to its clinical relevance and its role within the “inflammation-nutrition” framework. Unlike albumin, prealbumin possesses a shorter half-life, making it a more sensitive indicator of acute changes in nutritional reserves. In our UR group, significantly lower prealbumin levels likely signify a reduced physiological reserve, leaving patients vulnerable to infections and COPD exacerbations—the primary catalysts for unplanned readmissions.

### 4.4. Oncological Burden and Rationale for Model Selection

Oncological factors, specifically cancer stage, proved indispensable for accurate risk assessment. Stage III–IV disease was a significant predictor in the final GEE model (OR = 3.59, *p* = 0.014), underscoring its substantial clinical impact. Advanced-stage lung cancer is frequently associated with a higher tumor burden and necessitates more aggressive therapeutic regimens, which can exacerbate underlying COPD symptoms.

A critical consideration in this study was the selection of the final predictive model. While a model incorporating CRP and albumin yielded a higher statistical AUC, we prioritized the “age + cancer stage + SII + prealbumin” model. This decision was based on biological plausibility, as the coexistence of lung cancer and COPD involves a profound metabolic shift leading to “cancer-COPD cachexia.” Including prealbumin ensures the nomogram captures the “nutritional reserve” component, which is essential for identifying patients at risk of physiological decline. Furthermore, the statistical difference between the models was marginal when considering the holistic value. The chosen model also demonstrated good sensitivity (73.8%) and specificity (79.3%), offering a balanced tool for patient management.

### 4.5. Clinical Utility and Limitations

The proposed nomogram offers several practical advantages for clinical application. Firstly, it relies on laboratory parameters (SII and prealbumin) that are routinely obtained during standard admission, ensuring high feasibility without additional economic burden. Secondly, clinicians can use the nomogram at discharge to identify high-risk patients who may benefit from proactive strategies, such as personalized pulmonary rehabilitation, nutritional optimization, and more frequent post-discharge monitoring. For instance, a high-risk score could trigger a “Transition of Care” protocol, including a nutritional consult and an early post-discharge multidisciplinary clinic visit.

The proposed risk cutoff (e.g., a total score corresponding to a 25% predicted risk of UR) is intended as a clinical triage tool rather than a definitive diagnostic threshold. Patients identified as “High Risk” based on this cutoff could warrant the initiation of a multidisciplinary intervention bundle prior to discharge. This may include intensified pulmonary rehabilitation, optimization of bronchodilator therapy, and a mandatory follow-up teleconsultation within 72 h of discharge. While a lower threshold would increase sensitivity to capture more potential readmissions, the trade-off involves a higher burden on outpatient resources. Thus, the specific cutoff should be adaptable, allowing individual institutions to calibrate it based on their capacity for post-discharge monitoring and intervention.

Several limitations of this study warrant consideration. First, the cohort consisted exclusively of male cohort, reflecting the epidemiological reality that the prevalence of coexisting lung cancer and COPD is significantly higher in males within our specific regional demographic. However, the exclusion of female patients inherently limits the generalizability of our findings. Hormonal differences and varying smoking patterns between genders may influence both inflammatory profiles and respiratory outcomes; therefore, these results should be extrapolated to female populations with caution. Second, while median imputation was used for the minor missing prealbumin data (9.7%), and a sensitivity analysis suggested the data were missing at random, potential bias cannot be entirely excluded. Third, although the data are clustered within 22 patients, the use of GEE appropriately accounts for intra-patient correlation. A sensitivity analysis excluding the most extreme patient yielded consistent results (e.g., SII OR = 1.548, *p* = 0.001; AUC = 0.817), supporting the robustness of our model. Nonetheless, the small number of unique patients limits the generalizability, and external validation in larger cohorts is essential. Fourth, and most importantly, this study represents a model development and internal validation phase. Although internal validation via calibration plots and DCA demonstrated robust performance, these methods only assess model optimism within the derivation cohort. External validation in independent, multi-center cohorts is essential to confirm the model’s generalizability, predictive precision, and clinical utility across diverse geographic regions, ethnic populations, and clinical settings. Furthermore, as a single-center [30] retrospective study, the developed nomogram may be influenced by local clinical practices and patient demographics. To address this limitation, we are planning a prospective multi-center validation study across multiple institutions in different geographic regions. This will allow us to assess the model’s generalizability, evaluate its performance across diverse patient populations, and refine the nomogram based on heterogeneous clinical practices before considering its broader clinical implementations.

## 5. Conclusions

In summary, this study identified SII as a strong independent predictor of unplanned readmission in male patients with coexisting lung cancer and COPD. The developed nomogram, integrating age, SII, serum prealbumin, and cancer stage, provides a reliable and individualized risk-assessment tool during this model development phase. This “inflammation-nutrition-tumor” framework may help clinicians identify high-risk individuals for targeted interventions, with the goal of reducing readmission rates. External validation in prospective, multi-center cohorts is required before this tool can be recommended for widespread clinical implementation.

## Figures and Tables

**Table 1:** Clinical and laboratory characteristics of the episodes (*n* = 207).

Characteristics	Total (*n* = 207)	PR Group (*n* = 165)	UR Group (*n* = 42)	*p*-Value
**Demographics**				
Age (years) Median (IQR)	75 (69–78)	75 (69–78)	77 (71–78.3)	0.096
Smoking history *n* (%)	158 (76.3)	124 (75.2)	34 (81.0)	0.417
**Oncological Data**				
**Histology,** ***n*** **(%)**				0.591
- SCC	94 (45.4)	74 (44.8)	20 (47.6)	
- Adenocarcinoma	82 (39.6)	67 (40.6)	15 (35.7)	
- SCLC	31 (15.0)	24 (14.5)	7 (16.7)	
**Cancer Stage,** ***n*** **(%)**				**0.018**
- Stage I–II	115 (55.6)	100 (60.6)	15 (35.7)	
- Stage III–IV	92 (44.4)	65 (39.4)	27 (64.3)	
**COPD Severity,** ***n*** **(%)**				**0.031**
- GOLD I–II	159 (76.8)	132 (80.0)	27 (64.3)	
- GOLD III–IV	48 (23.2)	33 (20.0)	15 (35.7)	
**Laboratory Parameters**				
SII (10^9^/L), Median (IQR)	612.4 (396.1–1051.8)	504.9 (344.9–712.5)	1474.5 (1110.1–1939.8)	**<0.001**
CRP (mg/L), Median (IQR)	6.8 (2.5–16.8)	4.9 (2.1–10.4)	19.7 (10.9–34.1)	**<0.001**
Hemoglobin (g/L), Mean ± SD	128.4 ± 15.6	129.1 ± 15.2	125.6 ± 17.1	0.194
Albumin (g/L), Mean ± SD	37.6 ± 4.3	38.5 ± 3.9	34.3 ± 4.2	**<0.001**
Prealbumin (mg/L), Median (IQR)	193.0 (155.0–239.0)	201.0 (161.0–246.0)	159.5 (130.8–202.3)	**0.002**

**Abbreviations:** PR: Planned Readmission; UR: Unplanned Readmission; IQR: Interquartile Range; SCC: Squamous Cell Carcinoma; SCLC: Small Cell Lung Cancer; SII: Systemic Immune-Inflammation Index; CRP: C-reactive Protein; GOLD: Global Initiative for Chronic Obstructive Lung Disease; SD: Standard Deviation. **Notes:** Continuous variables are expressed as Mean ± SD (for normally distributed data, assessed by the Shapiro–Wilk test) or Median (IQR) (for non-normally distributed data). Comparison between groups was performed using the Independent-samples *t*-test, Mann–Whitney *U* test, or $\chi^{2}$ test. Bold values indicate statistical significance (*p* < 0.05).

**Table 2:** Univariable and multivariable GEE analysis for predictors of unplanned readmission.

Variables	Univariate Analysis OR (95% CI)	*p*-Value	Multivariable Analysis OR (95% CI)	*p*-Value
Age	1.061 (0.999–1.126)	0.055	1.048 (1.014–1.083)	0.005
SII (per 500-unit increment)	1.506 (1.154–1.964)	0.003	1.490 (1.234–1.798)	<0.001
CRP	1.057 (1.040–1.073)	<0.001	—	—
Serum Albumin	0.793 (0.696–0.903)	<0.001	—	—
Serum Prealbumin	0.927 (0.859–1.000)	0.050	0.950 (0.896–1.007)	0.087
Cancer Stage				
- Early (Stage I–II )	1.000 (Reference)	—	1.000 (Reference)	—
- Advanced (Stage III–IV )	5.223 (0.889–30.686)	0.067	3.590 (1.301–9.909)	0.014
COPD Severity				
- GOLD I–II	1.000 (Reference)	—	—	—
- COPD III–IV	2.116 (0.759–5.898)	0.152	—	—

**Abbreviations:** OR: Odds Ratio; CI: Confidence Interval. **Notes:** Only variables with *p* < 0.10 in univariate analysis were candidates for multivariable regression.

## Data Availability

The data presented in this study are available on request from the corresponding author due to ethical and privacy restrictions. The data are not publicly available because they contain information that could compromise the privacy of research participants.

## References

[1] Hendriks L.E.L., Remon J., Faivre-Finn C., Garassino M.C., Heymach J.V., Kerr K.M., Tan D.S.W., Veronesi G., Reck M. (2024). Non-small-cell lung cancer. Nat. Rev. Dis. Primers.

[2] de Oca M.M., Perez-Padilla R., Celli B., Aaron S.D., Wehrmeister F.C., Amaral A.F.S., Mannino D., Zheng J., Salvi S., Obaseji D. (2025). The global burden of COPD: Epidemiology and effect of prevention strategies. Lancet Respir. Med..

[3] Aaron S.D., Vandemheen K.L., Whitmore G.A., Bergeron C., Boulet L., Côté A., Mclvor R.A., Penz E., Field S.K., Lemière C. (2024). Early diagnosis and treatment of COPD and asthma—A randomized, controlled trial. N. Engl. J. Med..

[4] Qi C., Sun S.W., Xiong X.Z. (2022). From COPD to lung cancer: Mechanisms linking, diagnosis, treatment, and prognosis. Int. J. Chron. Obstruct. Pulmon. Dis..

[5] Zhou C., Qin Y., Zhao W., Liang Z., Li M., Liu D., Bai L., Chen Y., Chen Y., Cheng Y. (2023). International expert consensus on diagnosis and treatment of lung cancer complicated by chronic obstructive pulmonary disease. Transl. Lung Cancer Res..

[6] Zumbrunn A., Bachmann N., Bayer-Oglesby L., Joerg R., SIHOS Team (2022). Social disparities in unplanned 30-day readmission rates after hospital discharge in patients with chronic health conditions. PLoS ONE.

[7] Kan S.W., Yen H.Y., Chi M.J., Huang H.Y. (2025). Influence of physical function and frailty on unplanned readmission in middle-aged and older patients discharged from a hospital. Sci. Rep..

[8] Sharma Y., Mangoni A.A., Rao S., Batuwaththagamage I.K., Kaambwa B., Woodman R., Horwood C., Thompson C. (2025). Prevalence and characteristics of potentially avoidable unplanned readmissions: A retrospective cohort study. Aust. Health Rev..

[9] Myers L.C., Camargo C., Escobar G., Liu V.X. (2021). Expanding post-discharge readmission metrics in patients with chronic obstructive pulmonary disease. Chronic Obstr. Pulm. Dis..

[10] Krumholz H.M. (2013). Post-hospital syndrome--an acquired, transient condition of generalized risk. N. Engl. J. Med..

[11] Qu H., Zhu M., Shan C., Ji X., Ji G., Zhang W., Zhang H., Chen B. (2023). Prevalence, diagnosis, and treatment of chronic obstructive pulmonary disease in a hospitalized lung cancer population: A single center study. J. Thorac. Dis..

[12] Zhang R., Tan X., Chen Q., Wei J., Gai J., Wang Y., Yang Z., Li J., Zhu L., Huang Z. (2017). Investigation of lung cancer patients complicated with chronic obstructive pulmonary disease in thoracic surgical department. Zhongguo Fei Ai Za Zhi.

[13] Austin S.R., Wong Y.N., Uzzo R.G., Beck J.R., Egleston B.L. (2015). Why summary comorbidity measures such as the Charlson comorbidity index and Elixhauser score work. Med. Care.

[14] Sharma N., Schwendimann R., Endrich O., Ausserhofer D., Simon M. (2021). Comparing Charlson and Elixhauser comorbidity indices with different weightings to predict in-hospital mortality. BMC Health Serv. Res..

[15] Yang C.C., Fong Y., Lin L.C., Que J., Ting W.C., Chang C.L. (2018). The age-adjusted Charlson comorbidity index is a better predictor of survival in operated lung cancer patients than the Charlson and Elixhauser comorbidity indices. Eur. J. Cardiothorac. Surg..

[16] Grose D., Morrison D.S., Devereux G., Jones R., Sharma D., Selby C., Docherty K., McIntosh D., Nicolson M., McMillan D.C. (2015). The impact of comorbidity upon determinants of outcome in patients with lung cancer. Lung Cancer.

[17] Charison M.E., Carrozzino D., Guidi J., Patierno C. (2022). Charlson Comorbidity Index: A critical review of clinimetric properties. Psychother. Psychosom..

[18] Deng H., Zhu S., Yu F., Song X., Jin X., Ding X. (2024). Analysis of predictive value of cellular inflammatory factors and T cell subsets for disease recurrence and prognosis in patients with acute exacerbations of COPD. Int. J. Chron. Obstruct. Pulmon. Dis..

[19] Mazzella A., Orlandi R., Maiorca S., Uslenghi C., Chiari M., Bertolaccini L., Casiraghi M., Lo Iacono G., Girelli L., Spaggiari L. (2024). How general and inflammatory status impacts on the prognosis of patients affected by lung cancer: State of the art. Biomedicines.

[20] Hassanzadeh A., Allahdadi M., Nayebirad S., Namazi N., Nasli-Esfahani E. (2025). Implementing novel complete blood count-derived inflammatory indices in the diabetic kidney diseases diagnostic models. J. Diabetes Metab. Disord..

[21] Xu W., Liu X., Yan C., Abdurahmane G., Lazibiek J., Zhang Y., Cao M. (2024). The prognostic value and model construction of inflammatory markers for patients with non-small cell lung cancer. Sci. Rep..

[22] Ding H.P., Ling Y.Q., Chen W., Ding Q., Xu L.Q., Wu Y., Wang Q., Ni T.H., He B.Q. (2024). Effects of nutritional indices and inflammatory parameters on patients received immunotherapy for non-small cell lung cancer. Curr. Probl. Cancer.

[23] Iasonos A., Schrag D., Raj G.V., Panageas K.S. (2008). How to build and interpret a nomogram for cancer prognosis. J. Clin. Oncol..

[24] Tao M., Yan L., Lang Y., Shen L., Chen S., Cai N. (2025). Development and validation of a nomogram model for predicting the occurrence of necrotizing enterocolitis in premature infants with late-onset sepsis. Eur. J. Med. Res..

[25] Utsumi T., Ishitsuka N., Noro T., Suzuki Y., Iijima S., Sugizaki Y., Somoto T., Oka R., Endo T., Kamiya N. (2025). Development, validation, and clinical utility of a nomogram for urological tumors: How to build the best predictive model. Int. J. Urol..

[26] Chiu K.C., Lin W.C., Chang C.L., Wu S.Y. (2021). Impact of chronic obstruction pulmonary disease on survival in patients with advanced stage lung squamous cell carcinoma undergoing concurrent chemoradiotherapy. Cancers.

[27] Tian Z., Xi Y., Chen M., Hu M., Chen F., Wei L., Zhang J. (2025). Construction of a nomogram model for predicting pathologic complete response in breast cancer neoadjuvant chemotherapy based on the pan-immune inflammation value. Curr. Oncol..

[28] Zhao X., Zhao H., Dai K., Zeng X., Li Y., Yang F., Jiang G. (2025). Computed tomography-based radiomic nomogram to predict occult pleural metastasis in lung cancer. Curr. Oncol..

[29] Gandhi S., Chandna S., Chinnadurai V., Vidyarthi P. (2025). A novel serum inflammation risk-index (SIRI-RT)-driven nomogram for predicting secondary malignancy outcomes post-radiotherapy. Cancers.

[30] Baş O., Tokatlı M., Güdük N., Erdoğan D., Boyraz N.E., Çengelci Ç., Guven D.C., Dizdar Ö., Türker F.A., Aksoy S. (2025). Clinical characteristics and management of patients admitted to the supportive care clinic and predisposing factors of unplanned hospital readmission: Single-center experience. J. Clin. Med..

